# Supplementary figures and images for: A Common Missense Variant, W335S, in β2-Glycoprotein I (APOH) is Associated with Increased Autoantibody Levels but Reduced Venous Thromboembolism Risk
Source: medRxiv. 2026 Mar 5:2026.03.04.26347632. Preprint. [Version 1] doi: 10.64898/2026.03.04.26347632 (PMC13004100; doi:10.64898/2026.03.04.26347632)

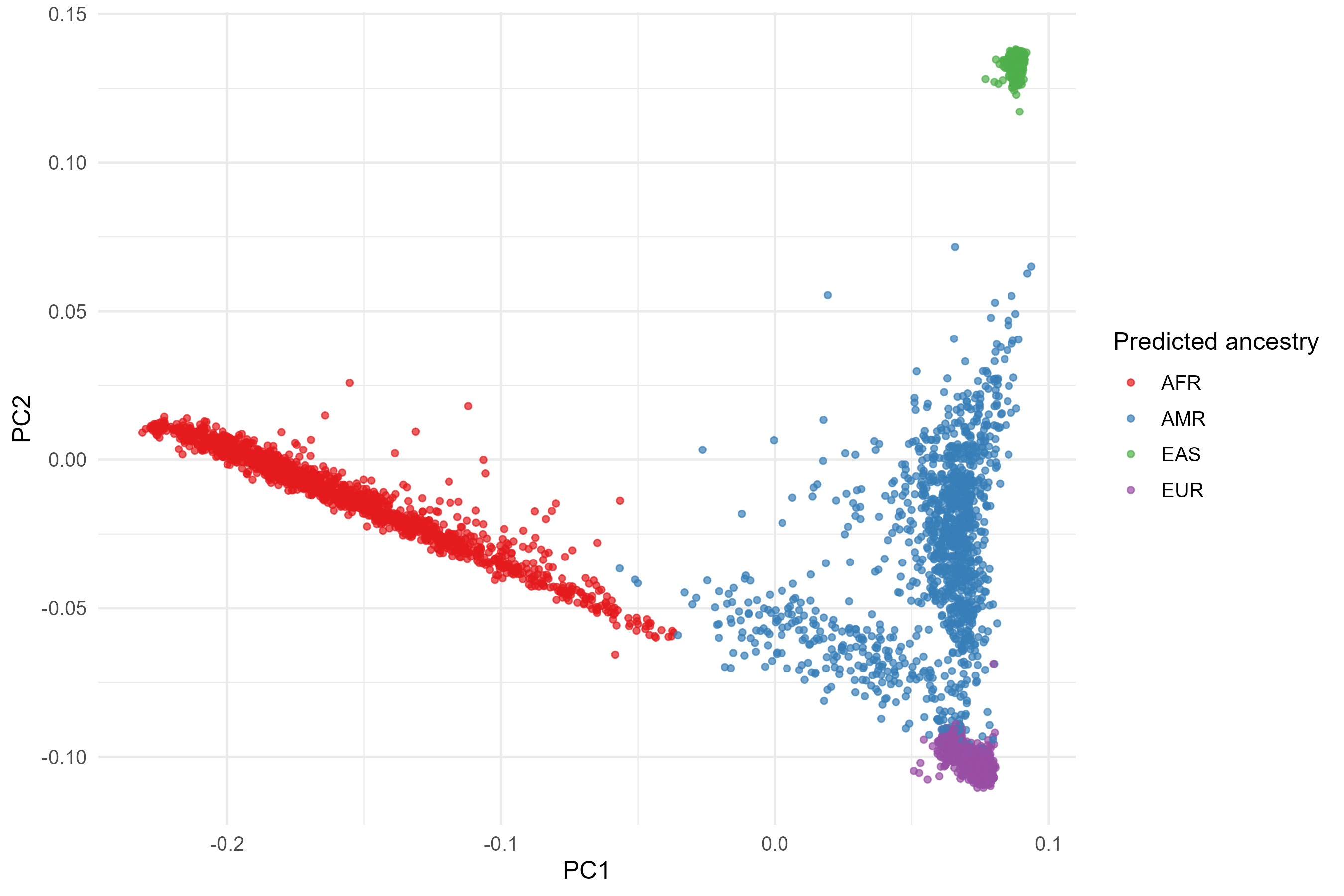

Supplement: Supplement 2 [file media-2.tif]

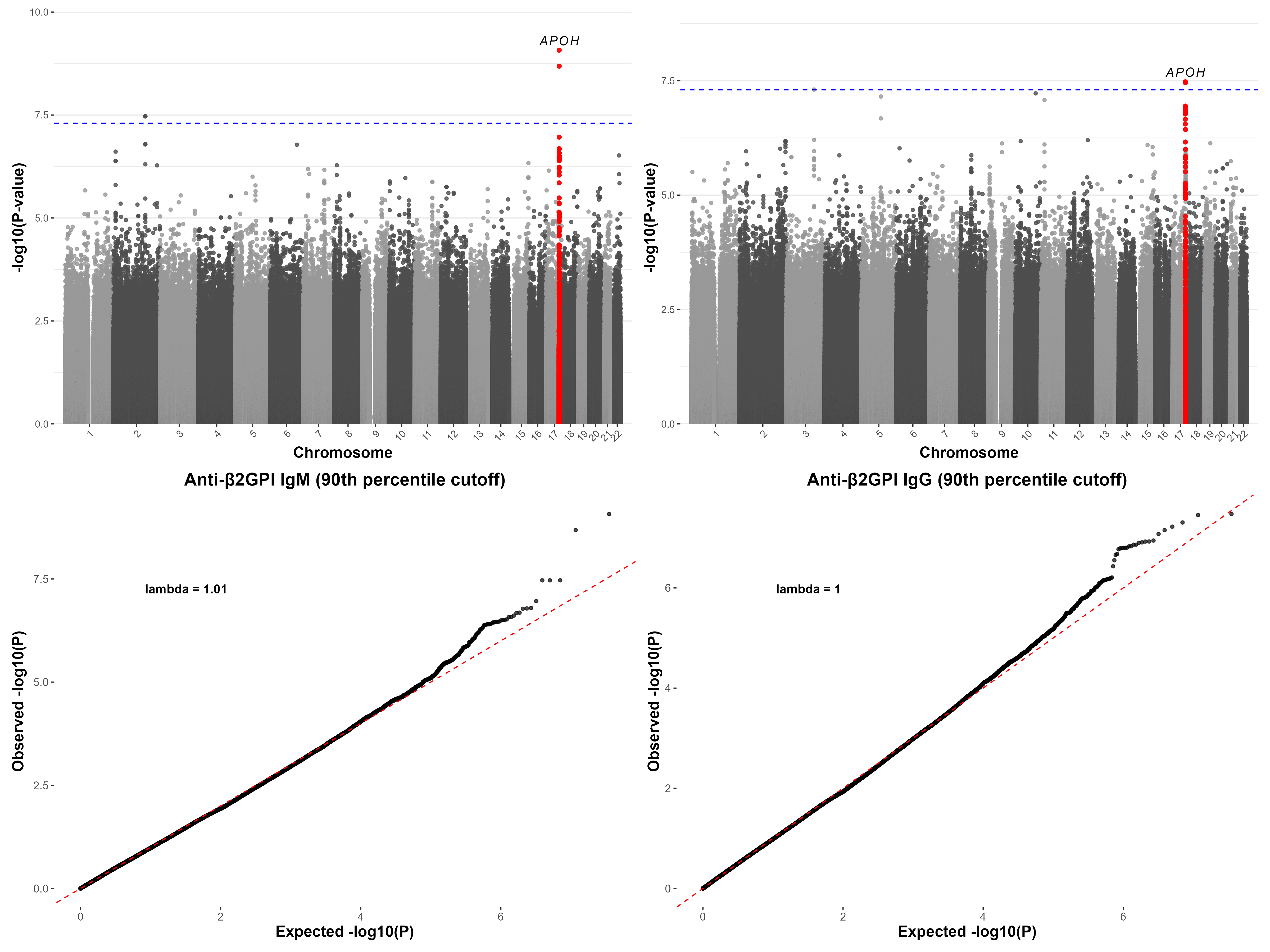

Supplement: Supplement 3 [file media-3.tif]

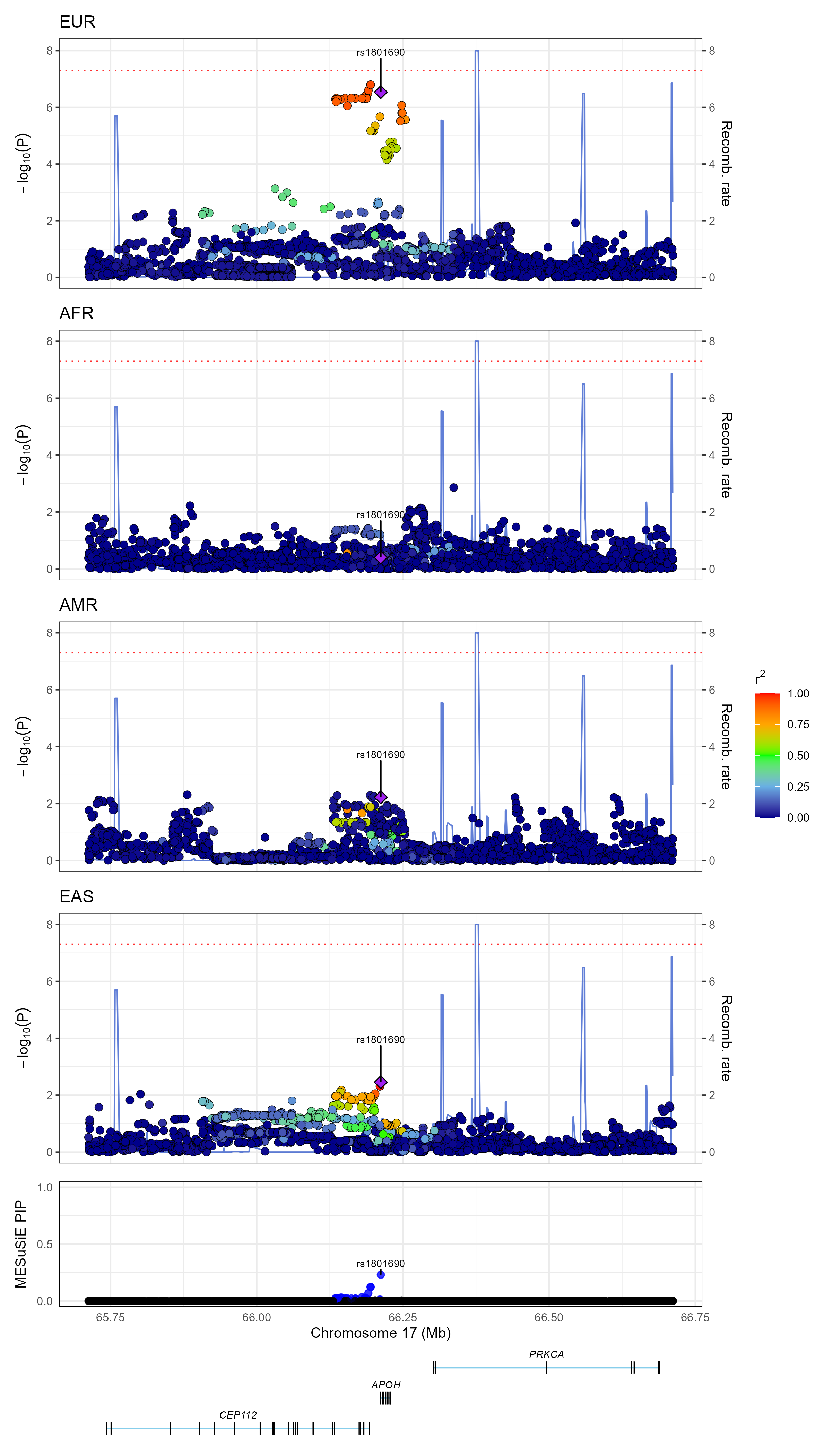

Supplement: Supplement 4 [file media-4.tif]

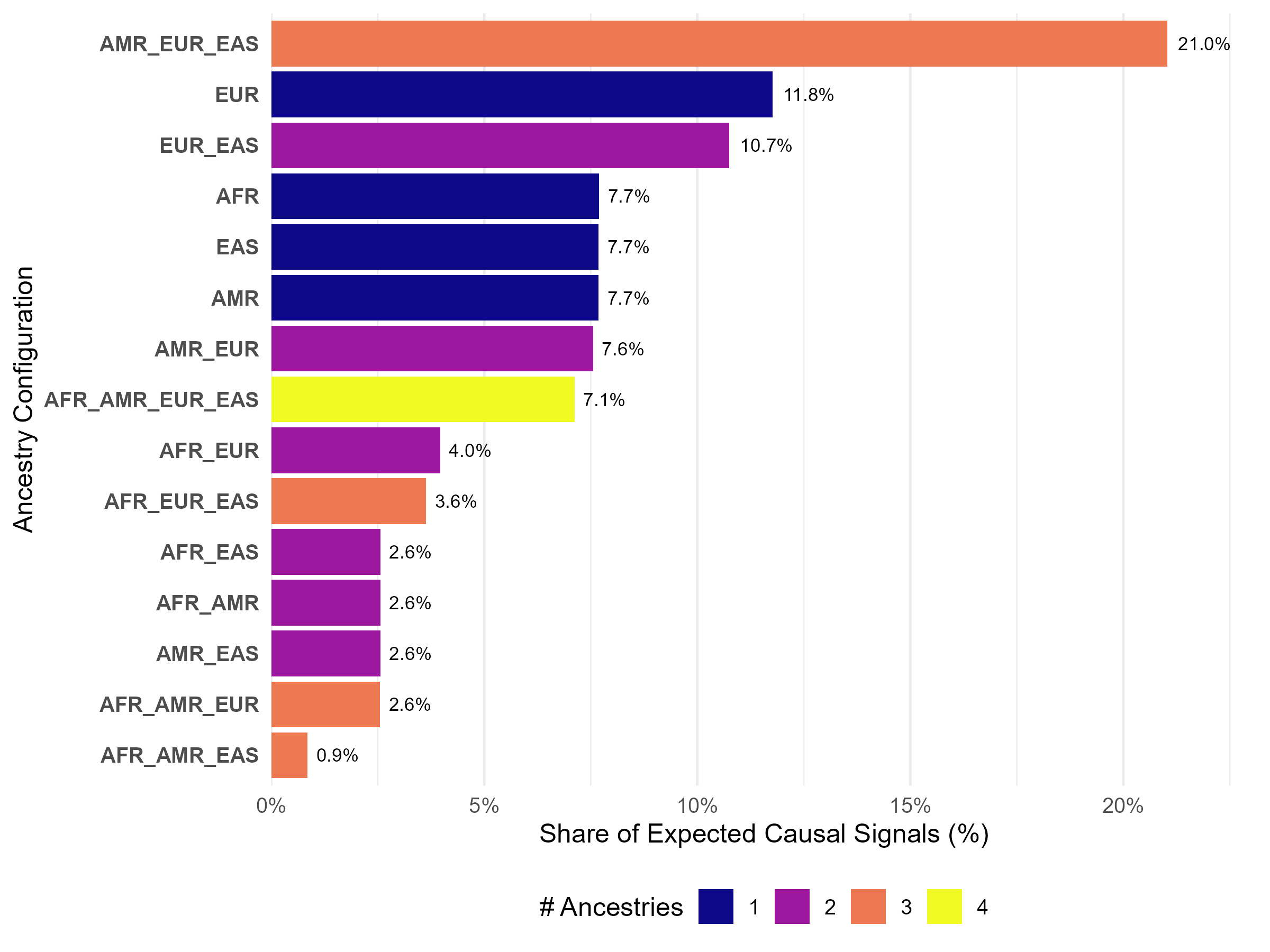

Supplement: Supplement 5 [file media-5.tif]

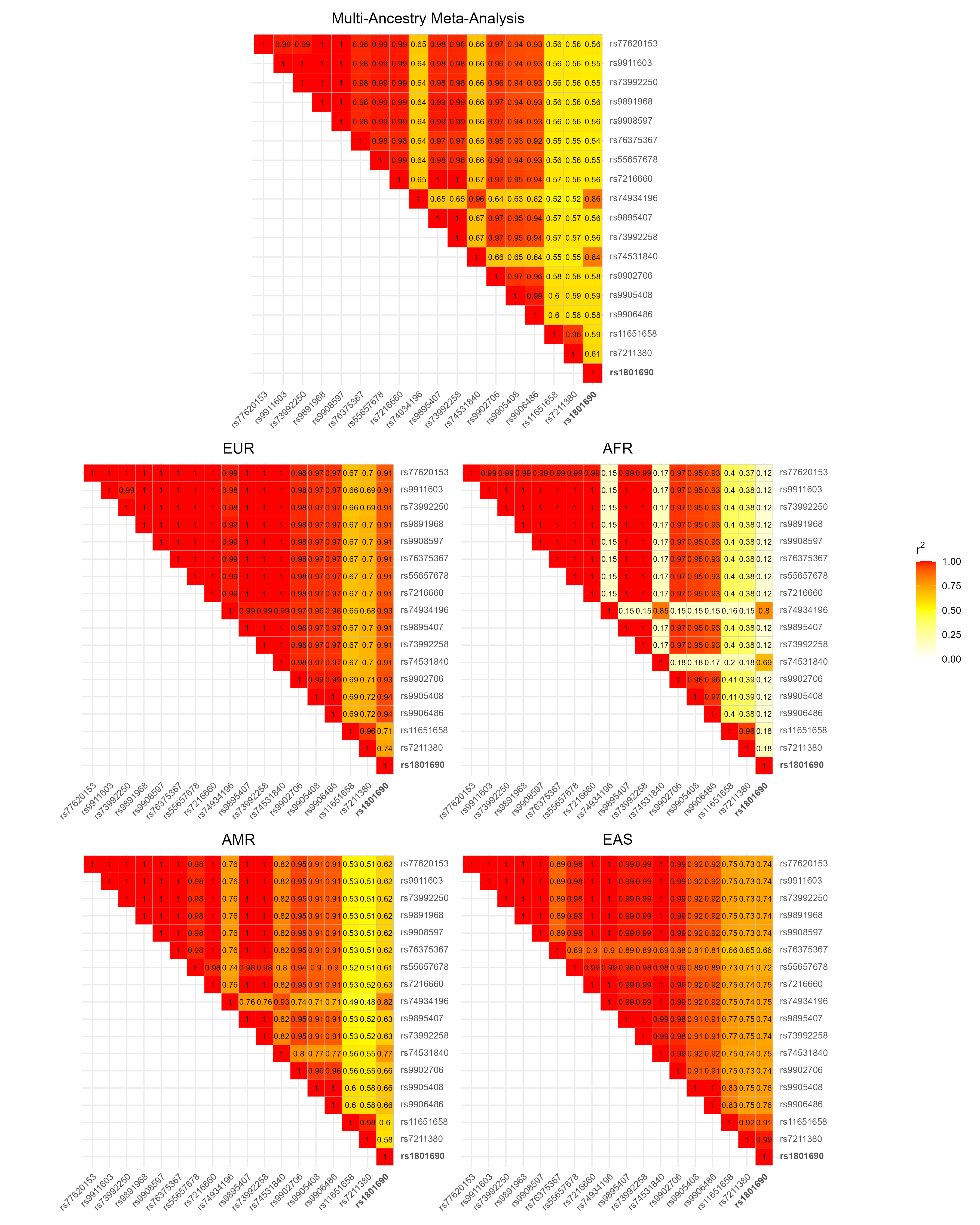

Supplement: Supplement 6 [file media-6.tif]

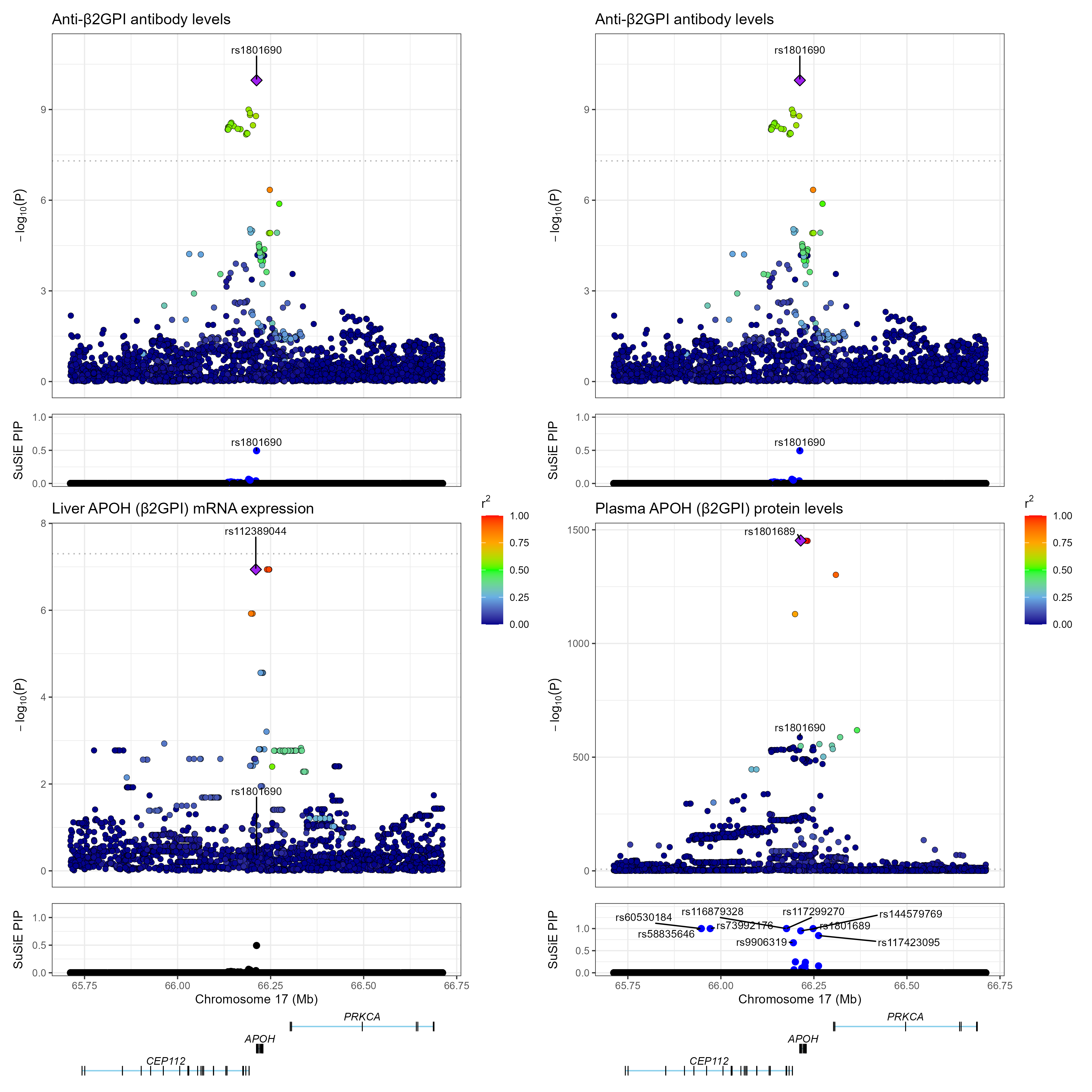

Supplement: Supplement 7 [file media-7.tif]
